# Mutually Coupled Time-to-Digital Converters (TDCs) for Direct Time-of-Flight (dTOF) Image Sensors ‡

**DOI:** 10.3390/s18103413

**Published:** 2018-10-11

**Authors:** Augusto Ronchini Ximenes, Preethi Padmanabhan, Edoardo Charbon

**Affiliations:** 1 AQUA Laboratory, Delft University of Technology (TU Delft), 2628 CD Delft, The Netherlands; 2 AQUA Laboratory, École Polytechnique Fédérale de Lausanne (EPFL), 2000 Neuchâtel, Switzerland; preethi.padmanabhan@epfl.ch (P.P.); edoardo.charbon@epfl.ch (E.C.)

**Keywords:** ring oscillator, clock distribution, synchronization, low-jitter TDC, dTOF image sensor, frequency synthesizer

## Abstract

Direct time-of-flight (dTOF) image sensors require accurate and robust timing references for precise depth calculation. On-chip timing references are well-known and understood, but for imaging systems where several thousands of pixels require seamless references, area and power consumption limit the use of more traditional synthesizers, such as phase/delay-locked loops (PLLs/DLLs). Other methods, such as relative timing measurement (start/stop), require constant foreground calibration, which is not feasible for outdoor applications, where conditions of temperature, background illumination, etc. can change drastically and frequently. In this paper, a scalable reference generation and synchronization is provided, using minimum resources of area and power, while being robust to mismatches. The suitability of this approach is demonstrated through the design of an 8×8 time-to-digital converter (TDC) array, distributed over 1.69 mm^2^, fabricated using TSMC 65 nm technology (1.2 V core voltage and 4 metal layers—3 thin + 1 thick). Each TDC is based on a ring oscillator (RO) coupled to a ripple counter, occupying a very small area of 550 μm^2^, while consuming 500 μW of power, and has 2 μs range, 125 ps least significant bit (LSB), and 14-bit resolution. Phase and frequency locking among the ROs is achieved, while providing 18 dB phase noise improvement over an equivalent individual oscillator. The integrated root mean square (RMS) jitter is less than 9 ps, the instantaneous frequency variation is less than 0.11%, differential nonlinearity (DNL) is less than 2 LSB, and integral nonlinearity (INL) is less than 3 LSB.

## 1. Introduction

Direct time-of-flight (dTOF) imaging is a depth sensing technique [1] capable of providing fast and accurate distance measurements over a large range of distances. Although different approaches can be used to implement a dTOF sensor, including time-gated quanta image sensors [2] and single-shot measurements using silicon photomultiplers (SiPMs) [3], the most common and robust technique is based on time-correlated single-photon counting (TCSPC) [4] using time-to-digital converters (TDCs), which allows the system to be robust to background noise while detecting relatively weak signals. It consists of measuring the travel time of photons, known as time-tagged time-resolved (TTTR) [5], generated by a periodic light source such as a pulsed laser and accumulated into certain statistics, such as histograms of photon counts versus time. The system is capable of obtaining the target under strongly negative signal-to-noise ratio (SNR) regime [6], where the signal is the average number of photon events, correlated to the system’s light source, reflected from the target and detected by the sensor. The noise is the total dark-count and background illumination noise events.

The possibility of using a mass-produced technology such as the complementary metal–oxide–semiconductor (CMOS) for these systems has enabled many applications of dTOF image sensors. The potential is vast in consumer electronics such as augmented and virtual reality (AR/VR), biomedical imaging (e.g., positron emission tomography (PET) [7] and fluorescence lifetime imaging microscopy (FLIM) [8,9,10]), robotics, and most recently, light detection and ranging (LiDAR) for advanced driver-assistance systems (ADASs) and autonomous vehicles (AVs) [11].

Since dTOF operates by measuring the travel times of photons (absolute time interval), its performance depends directly on the ability to measure it accurately and quickly on-chip. The quality of the timing reference defines the accuracy of the measurement, so a power-efficient, robust, and scalable timing solution is highly desirable. Moreover, in large sensor arrays, where IR-drop and temperature drifts can cause resolution variation, degrading the timing information, dynamic performance variations must be considered in the design strategy in order to maximize the uniformity of the measurement.

In this paper, we propose a simple and scalable timing solution for dTOF image sensors, based on a shared-TDC architecture. The paper is organized as follows: Section 2 presents a shared topology and its impact on power consumption and photon detection saturation, in comparison to other approaches. Section 3 presents our conceptual solution for timing and synchronization, including analysis and simulation. The experimental results are presented and discussed in Section 4. Conclusions are drawn in Section 5.

## 2. TDC Sharing

### 2.1. Power Consumption

There are many ways to obtain a precise timing reference on silicon. The most common is by implementing a feedback system such as phase-locked loop (PLL) or delay-locked loop (DLL) [12], capable of frequency or delay scaling, synchronized to an off-chip crystal oscillator. Although several oscillator topologies exist, PLLs/DLLs are typically based on an inductor-capacitor (LC) tank, a ring oscillator (RO), or a relaxation oscillator. LC-tank oscillators are typically used in low-jitter PLLs, where their higher quality factor (Q) offers more precise timing compared to RO or relaxation oscillators [13]. However, their use in imagers is limited due to area constraints. RO-based PLLs/DLLs are preferred over relaxation oscillators due to their superior jitter performance and lower area, thus being the most suitable for imagers.

In dTOF imagers, time measurement is generally obtained through two methods. The first consists of implementing a TDC per pixel, operating in start–stop mode [4]. In the second method, a continuously running PLL provides a global reference signal for the sensor, serving directly as TDC and/or as reference for local interpolation TDCs [14,15]. Each method offers distinct advantages with respect to power consumption and conversion rate, and they will be evaluated next.

**Figure 1:**
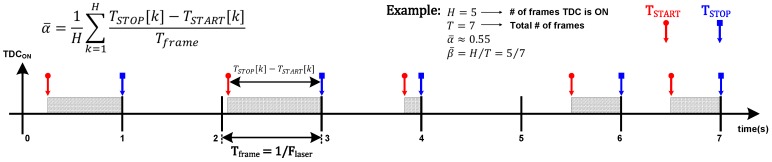
Time diagram example of a single time-to-digital converter (TDC) in event-driven mode.

Figure 1 introduces the concepts used throughout this paper. The first parameter, $\bar{\alpha }$, provides the average time for which a particular TDC stays activated. For example, in a noiseless system, $\bar{\alpha }$ would assume a value that corresponds roughly to the location of the target with respect to the time frame. In a noisy environment, it might assume a value closer to the middle point ($\bar{\alpha }\approx 0.5$), which is the average value of a uniformly distributed variation (since the noise is uncorrelated to the time frame). The presence of the signal might shift $\bar{\alpha }$ from the middle point, depending on its intensity compared to the noise. The second parameter, $\bar{\beta }$, is the average activity rate of one pixel, normalized to the laser frequency ($F_{laser}$). If the TDC is activated in all time frames, $\bar{\beta }$ is one. Otherwise, it assumes a value that indicates how often a TDC is used.

Another interpretation of $\bar{\alpha }$ and $\bar{\beta }$ is that their product indicates the duty cycle of a TDC, so its power consumption can be calculated. In this short observation shown in Figure 1, the TDC duty cycle is about 39.3% ($\bar{\alpha }\cdot \bar{\beta }$), although longer observation would be required to obtain such parameters. To be more generic, including the possibility of sharing a single TDC with multiple pixels (*M*), its duty cycle can be written as $\bar{\alpha }\cdot min\left(\bar{\beta }\cdot M,1\right)$, where any pixel could start the TDC, to a limit of activity equal to one.

**Figure 2:**
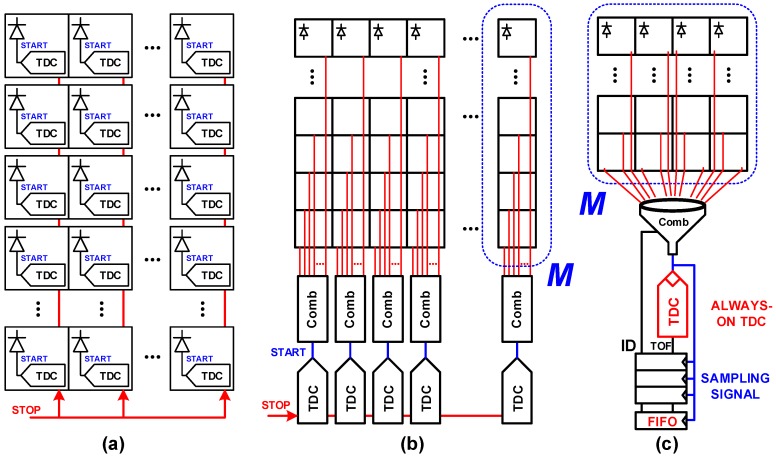
TDC arrangement. (**a**) Per-pixel, event-driven TDC; (**b**) Column-wise shared TDC; (**c**) Continuously running, shared TDC concept. FIFO: first-in-first-out; TOF: time-of-flight.

A generic power consumption required by the timing generation and acquisition is derived in (A4), and it is reproduced by:

(1) $$\begin{array}{cc} P_{T}=P_{PLL}+\#p\cdot C_{line}\cdot V^{2}\cdot F & +\bar{\alpha }\cdot P_{TDC}\cdot N\cdot min\left(\bar{\beta }\cdot M,1\right)\\ & +E_{comb}\cdot N\cdot min\left(\bar{\beta }\cdot M\cdot F_{laser},\tau^{-1}\right), \end{array}$$

where $P_{PLL}$ is the PLL power consumption. The second term refers to the dynamic power consumed in the distribution of multiple (#p), high-frequency (*F*) PLL phases, over capacitive wires ($C_{line}$), with voltage swing *V*. *N* is the total number of TDCs and *M* the number of pixels sharing a single TDC (M×N is the total number of pixels in the sensor). A combination circuit is necessary in the case of sharing structures, so the events in multiple pixels can be processed by the TDC, as sketched in Figure 2b,c. Thus, $E_{comb}$ is the energy consumed per event by such a combination circuit. τ is defined by the dead time of the combination circuit, limiting the activity among *M* pixels, and it will be discussed in Section 2.2. For a more direct comparison between both architectures, the power consumed by the PLL and in the distribution of its phases will be ignored.

The TDCs can operate in two different modes: event-driven or sampled (continuously running TDC). In per-pixel TDC (Figure 2a), the TDCs typically operate in event-driven mode, turning on upon a photon event, and stopping by the end of the time frame [4]. In this case, $E_{comb}$ from (1) can be neglected (since the pixel is connected directly to the TDC), so the total power over *M* pixels reduces to:

(2) $$P_{T,per-pixel}=\bar{\alpha }\cdot P_{TDC}\cdot M\cdot min\left(\bar{\beta },1\right),$$

where the number of pixels sharing a TDC is one and *N* is replaced by *M* to account for the total power over *M* pixels (*M* TDCs). Shared structures such as in Figure 2b,c can operate either in event-driven or sampled modes. For the event-driven mode, the power consumption of *M* pixels reduces to:

(3) $$P_{T,shared\_event-driven}=\bar{\alpha }\cdot P_{TDC}\cdot min\left(\bar{\beta }\cdot M,1\right)+E_{comb}\cdot min\left(\bar{\beta }\cdot M\cdot F_{laser},\tau^{-1}\right).$$

It is important to observe that shared architectures that operate in event-driven mode are only viable for photon-starved regimes, or in a scanning mode where at each point in time the TDC is not effectively shared, but dedicated to a single pixel [16] or operates as a SiPM [17]. For this reason, this mode will not be considered further in this paper.

For the sampled approach, a continuously running TDC is shared among several pixels, as shown in Figure 2b,c, depending on the requirements of power consumption and conversion rate, which will be seen further. In contrast to an event-driven approach, upon an event in any of these pixels, the TDC samples a time, that is, a timestamp is created and streamed through a first-in-first-out (FIFO) bus, along with the address of the detecting pixel. Multiple events can occur among those pixels, where the conversion time for the TDC itself is negligible, and the system saturation depends largely on the combination logic dead time.

Thus, from (1), the overall power consumption, related to the timing of *M* pixels, is given by:

(4) $$P_{T,shared\_sampled}=P_{TDC}+E_{comb}\cdot min\left(M\cdot F_{laser}\cdot \bar{\beta },\tau^{-1}\right),$$

where the first term is due to a continuously running TDC, and the second term is due to the combination circuit power. It is relevant to observe that, independent of the activity ($\bar{\beta }$) or number of pixels sharing a TDC (*M*), the TDC stays on all the time, which indicates that its power consumption is at its maximum, yet constant. By separating the power grid that connects the always-on TDC (s), a constant power consumption is expected. Thus, a constant IR-drop is also expected, even though the overall power ($P_{T,shared\_sampled}$) can vary with activity, which can be provided by a different power line.

Evidently, in the case where a PLL is present, the power consumption would be higher, as would be the precision, introducing new quality variables into the comparison. Nevertheless, by analyzing (2) with (4), it is possible to obtain the following condition (A5):

(5) $$\begin{array}{cc} P_{T,per-pixel} & ⩾P_{T,shared\_sampled},\\ M & ⩾\frac{1}{\bar{\alpha }\cdot min\left(\bar{\beta },1\right)-\left(\frac{E_{comb}\cdot min(F_{laser}\cdot \bar{\beta },{\left(M\cdot \tau \right)}^{-1})}{P_{TDC}}\right)}. \end{array}$$

For the shared approach to offer better power efficiency than pure event-driven systems, the number of pixels sharing a single TDC, *M*, should satisfy (5).

### 2.2. Effects on Sensitivity

The main drawback of sharing topologies is the inevitable chance of event collisions—specifically for signal photons, since they are close in time. The timing response of a target is a combination of the laser pulse width and the target depth variation. The target shape, the amount of pixels sharing a TDC, the arrangement of these pixels (in a square or rectangle, in a column, in a row, etc.), and the laser pulse width will influence the collision probability in the combination circuit. Thus, to evaluate the sensitivity reduction of the sharing case, $\bar{\beta }$ can be modified following a non-paralyzable model [18,19] that evaluates the probability of multiple event occurrences within the combination circuit dead time which are not recorded, obtaining the effective average activity rate per pixel, such as:

(6) $${\bar{\beta }}_{shared}=\frac{\bar{\beta }}{1+M\cdot 1/T_{win}\cdot \bar{\beta }\cdot \tau },$$

where $T_{win}$ is the observation window and τ the combination circuit dead time. In the arrangement of Figure 2c, $\tau =\Delta t_{comb}\cdot \log_{2}M$, where $\Delta t_{comb}$ is the delay of each binary combination stage. All the uncertainties that would influence the timing response of the target can be incorporated into $T_{win}$, such as the laser pulse width and the target shape. The sensor saturation and also the maximum conversion rate of shared topologies is defined by the dead time of the combination circuit. This implies that the combination circuit is “reset” after one event and is readily available for a new detection, whereas the TDC dead time is negligible, since it is just sampled. If the combination circuit is composed of simple logic gates, then τ must also account for the pixels’ outputs pulse widths, which might require monostable generators in order to avoid excessive sensitivity degradation [20].

Although per-pixel TDCs do not suffer from the aforementioned saturation because each pixel is independent, the influence of noise can blind the pixels for the signal by occupying the TDCs with noise events early on in the time frame. Moreover, in conditions where background illumination is high (indoor/outdoor applications) and the probability of detecting noise is much higher than signal [6], TCSPC operation [21] is generally needed, requiring higher statistics that event-driven architectures would take longer to provide. This way, in order to evaluate sensitivity, two different components should be analyzed: the effective average activity rate, limited by the dead time and observation window, and the maximum conversion rate.

As an example, if the following parameters are used: $F_{laser}$ = 1 MHz, for 150 m LiDAR measurement, and $\bar{\alpha }\approx$ 50% (0.5), since the target and/or background noise can arrive anytime within the measurement window (for ultra high background noise, $\bar{\alpha }\to$ 1), the power consumption from the combination circuit can be estimated by the switching of $\log_{2}M$ capacitors (rough estimation of ∼1 fF per gate), in case of a simple OR-tree, thus $E_{comb}\approx 2\cdot \left(1/2\cdot C\cdot V^{2}\right)\cdot \log_{2}M$.

**Figure 3:**
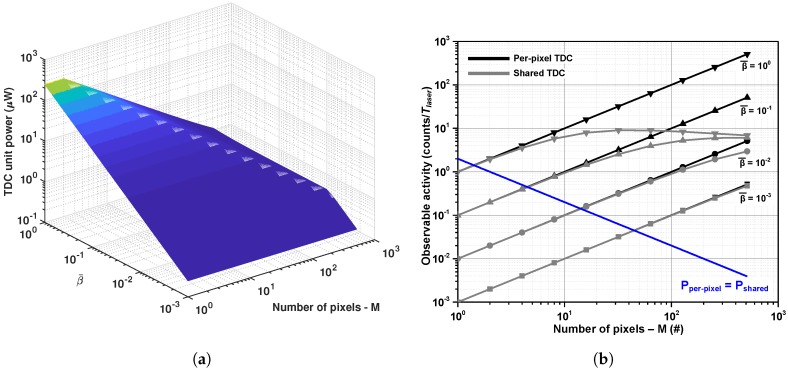
Relationship between power consumption, activity, and number of pixels. (**a**) Average power per TDC unit; (**b**) $\bar{\beta }$ compression due to combination dead time, within a laser pulse ($T_{laser}$) of 5 ns. Conditions above the blue line makes it more power-efficient to share a TDC instead of using a single TDC per pixel.

For a typical TDC power consumption of 500 μW [22], the relation between power, number of pixels sharing a TDC, and the activity $\bar{\beta }$, is plotted in Figure 3a. Figure 3b presents the maximum observable activity when a signal width ($T_{win}$) of 5 ns (75 cm, as a combination of laser pulse width and target variation) and a dead time $\Delta t_{comb}$ of 80 ps are used (arbitrary value: shorter for a simple logic gate; longer for a flip-flop, in 65 nm CMOS technology, for example). The observable activity relates to the maximum number of detectable events per laser pulse, based on $\bar{\beta }$ and *M* pixels. Since the event-driven approach can detect only a single event per time frame, the observable activity is the product of $\bar{\beta }$ and *M* (black curves). However, for the shared approach, with continuously running TDC (column-wise or in a different arrangement), the inevitable dead time required by the combination circuit limits the maximum observable activity (gray curves). Intuitively, the more a single TDC is shared, the lower the power per pixel, but the fewer photons the system can detect (for short observation). For long observations, the conversion rate of the proposed method is inversely proportional to the dead time of the combination circuit, which can reach Gtimestamps/s (per *M* pixels), whereas for the per-pixel TDC, the maximum conversion rate is still limited to $F_{laser}$ timestamps/s per pixel.

A LiDAR system typically operates under low detection probability, unless it has a very narrow field of view (FOV), high-intensity laser, or is used for short ranges. According to (6), for the system conditions mentioned previously and $\bar{\beta }$ of about 10% (0.1) (the signal probability is also about 10%), for a group of five or more pixels, it is more power efficient to share a single TDC than to have a per-pixel TDC. If 64 pixels share a single TDC [22] instead of 5, the power of such an arrangement is 3.2× lower than that of a per-pixel TDC. However, it is only able to detect 62% of photons for the 5 ns $T_{win}$ (see Figure 3). If the maximum conversion rate is considered (for activities not related to the laser itself, such as background light), the shared case is capable of 2 Gtimestamps/s (inverse of the combination circuit dead time, where τ = 80 ps$\cdot \log_{2}64$) for the group of 64 pixels, or, on average, 32 Mtimestamps/s/pixel. Meanwhile, for a per-pixel approach, only a single conversion per time frame (1 μs) is possible, and thus a maximum of 1 Mtimestamps/s/pixel. The choice between shared or per-pixel TDC will depend on the system. For LiDAR, where high background noise is often present, increasing throughput at lower power is essential, favoring the shared approach.

In conclusion, event-driven operation is the most power-efficient solution for photon-starved scenarios, where column-wise topologies operating in such conditions can offer even better power efficiency and precision, although it offers lower conversion rate per pixel, especially for short bursts of photons (i.e., in laser pulse width). Our proposed shared structure takes advantage of 3D-stacking technology, offering better power efficiency and higher conversion rate when the activity in the sensor increases, as well as better silicon utilization (more area for on-chip signal processing and storage), enabling more intelligent sensors. Moreover, column-wise approaches can also be shared and operate continuously, where the TDC array could be coupled linearly (instead of in two dimensions, as proposed), and it is a viable alternative for monolithic implementations, where it benefits from the same advantages discussed in our proposed approach.

For these reasons, we propose a sharing architecture for single-photon avalanche diode (SPAD) arrays in LiDAR. Furthermore, a technique for TDC synchronization will be discussed next, which is the best compromise for power and performance in the photon illumination regimes encountered in LiDAR.

## 3. Synchronization

Apart from providing a power-efficient timing reference throughout the sensor, it is essential to maintain a well-known and stable resolution, independent of mismatches and process-voltage-temperature (PVT) variations. Activity-dependent systems, where power consumption varies with incoming light (e.g., in event-driven approaches), are typically hard to predict and constant foreground calibration is required. In our proposed architecture, where the TDC power consumption is constant, as seen in Section 2.1, this is less of an issue. However, such designs are still subject to mismatch and PVT variations.

Thus, our proposed approach exploits the availability of continuously running oscillators by operating them mutually coupled, through a single phase, in a process of injection-locking at the fundamental frequency. When combined, the oscillators provide a much lower phase noise, while operating synchronously (phase/frequency locking), even under potential oscillator mismatches, without any external circuit or additional power consumption. Then, a single PLL can be implemented (using any node of the array as reference for the feedback path) to track PVT variations.

**Figure 4:**
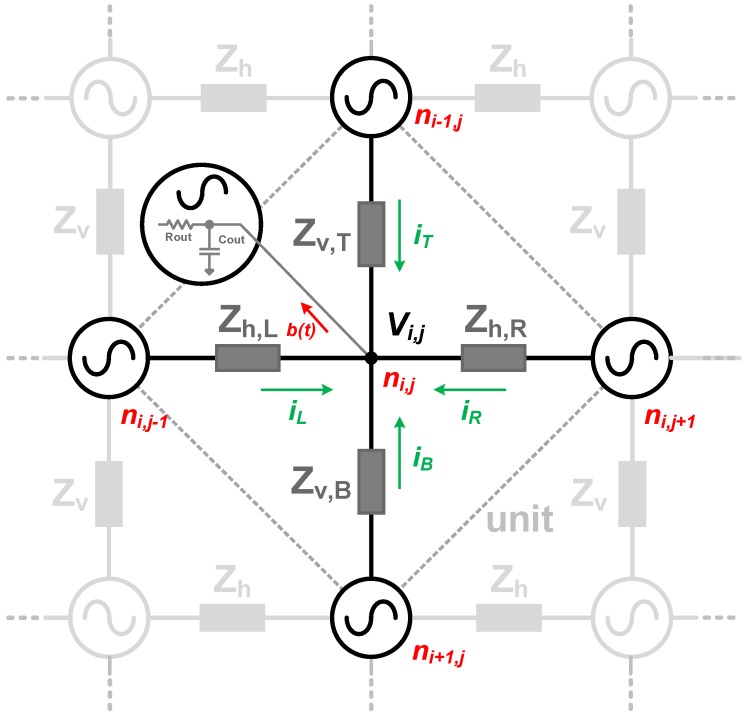
Generic mutually coupling oscillators concept.

**Figure 5:**
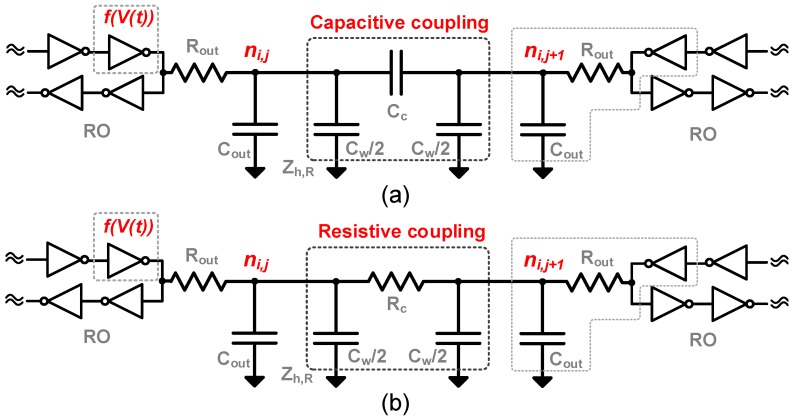
(**a**) Capacitive and (**b**) Resistive coupling elements between two generic ring oscillators (ROs) (only $Z_{h,R}$ shown).

The concept is shown in Figure 4, where the minimum cell is highlighted. The coupling elements are represented by $Z_{h,L}$, $Z_{h,R}$, $Z_{v,T}$, and $Z_{v,B}$ for the connecting impedances. The oscillators are based on ROs, where capacitive and resistive coupling are studied, as depicted in Figure 5. Inductive coupling was not considered due to practical layout implementations, and the parasitic inductance of the wire was neglected due to relatively low operation frequency and short length.

### 3.1. Non-Linear Modeling

Injection locking has been successfully used in many applications, such as high-frequency clock division [23], quadrature generation [24], clock distribution [25], etc. The effect has been extensively studied by several authors, based mostly on the generalized Adler’s equation [26,27], and the scope of this paper does not permit the physics of the process to be further revisited. Instead, we intend to provide a useful tool to design dTOF image sensors.

The dynamics of the system can be analyzed by performing a nodal analysis on the model shown in Figure 5. The process of synchronization occurs by injection-locking through the fundamental frequency, at a single node of each oscillator. The strength of the coupling element and the quality factor (Q) of the oscillator will define the maximum injection bandwidth, settling time, and sensitivity to neighboring disturbances, which depends on the target application and will be discussed further.

A non-linear phase macromodel is used to investigate the injection phenomenon [28]. The ROs dynamics are solved through ordinary differential equations at node $n_{i,j}$, shown in Figure 4, under the influence of its neighboring oscillators, at nodes $n_{i-1,j}$, $n_{i+1,j}$, $n_{i,j-1}$, $n_{i,j+1}$, and extrapolating it to the entire system. The numerical analysis of the perturbations is based on the Floquet theory of periodically time-varying systems [29] of ordinary differential equations.

The steady state voltage response of an oscillator, in the absence of any perturbation, can be represented by the time-dependent function $V_{s}\left(t\right)$. Under an external perturbation, b(t), the RO response becomes:

(7) $$V_{\left(i,j\right)}=V_{s}\left(t+\alpha \left(t\right)\right)+y\left(t\right),$$

where the term α(t) is the phase deviation caused by the disturbance b(t). The perturbation b(t) in this model is represented by currents from the neighboring oscillators $i_{L}$, $i_{R}$, $i_{T}$, $i_{B}$, as shown in Figure 4. The term y(t) is the orbital deviation reflecting any gain error, in the presence of this external perturbation. However, this term will not be considered for further analysis, as amplitude variations are negligible and the effect of the injection mechanism on the phase of the oscillator is dominant [28]. Thus, the perturbed steady state solution can be approximated by $V_{s}\left(t+\alpha \left(t\right)\right)$.

A current analysis of the capacitive coupling, shown in Figure 5a, at node $n_{i,j}$, can be obtained by:

(8) $$\begin{array}{cc} \frac{dV_{\left(i,j\right)}}{dt}= & \frac{f(V(t))}{R_{out}\left(C_{out}+2C_{w}+4C_{c}\right)}-\frac{V_{\left(i,j\right)}}{R_{out}\left(C_{out}+2C_{w}+4C_{c}\right)}\\ + & \frac{C_{c}}{\left(C_{out}+2C_{w}+4C_{c}\right)}\cdot \frac{d}{dt}\left(V_{\left(i+1,j\right)}+V_{\left(i-1,j\right)}+V_{\left(i,j+1\right)}+V_{\left(i,j-1\right)}\right), \end{array}$$

where $V_{\left(i,j\right)}$ is the nodal voltage, and $R_{out}$ and $C_{out}$ are defined by the RO output impedance. $C_{w}$ is the shunt parasitic capacitance from the coupling line, and $C_{c}$ is the effective coupling capacitance. The term f(V(t)) models the RO stage non-linearity for the delay stage preceding the coupled node by a hyperbolic tangent function, $tanh(G_{m}V\left(t\right))$, where $G_{m}$ is the large-signal stage transconductance.

Similarly, in the case of a resistive coupling element (Figure 5b), the voltage at node $n_{i,j}$ is given by:

(9) $$\begin{array}{cc} \frac{dV_{\left(i,j\right)}}{dt}= & \frac{f(V(t))}{R_{out}\left(C_{out}+2C_{w}\right)}-\frac{V_{\left(i,j\right)}}{R_{out}\left(C_{out}+2C_{w}\right)}\\ + & \frac{V_{\left(i+1,j\right)}+V_{\left(i-1,j\right)}-2V_{\left(i,j\right)}+V_{\left(i,j+1\right)}+V_{\left(i,j-1\right)}-2V_{\left(i,j\right)}}{R_{c}\left(C_{out}+2C_{w}\right)}. \end{array}$$

Equations (8) and (9) were numerically solved in MATLAB for TDC networks of 4 × 4, 8 × 8, and 16 × 16, using seven-stage ROs, although the modeling holds true for any number of RO stages, just with an impact on its dynamics. The networks are terminated (at their boundaries) by the same coupling element, but open at one of its ends.

For the following simulation, the parameters $R_{out}$, $C_{out}$ and $G_{m}$ (refer Figure 5) were chosen (based on typical values) to obtain an average oscillation period of 2 ns (500 MHz). Random mismatches were also included, impacting on about ±15% period variation among the oscillators, in order to verify the robustness of the method.

**Figure 6:**
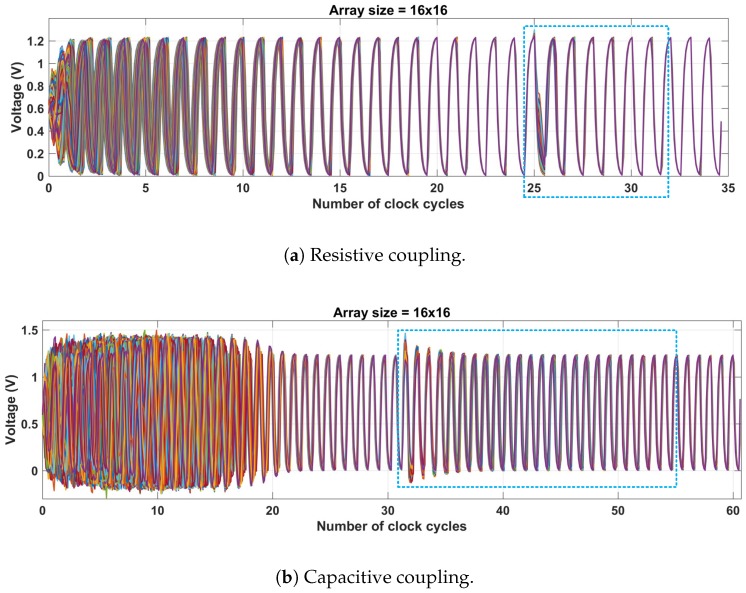
Voltage waveforms of a 16×16 coupled RO network under ±15% random initial conditions and with disturbance introduced in 32 ROs in the case of (**a**) resistive coupling with $R_{c}$ = 250 Ω and (**b**) capacitive coupling with $C_{c}$ = 240 fF.

The steady state voltage for a 16 × 16 RO array, using coupling resistance $R_{c}$ = 250 Ω, is shown in Figure 6a. The ROs started with a random period of 2 ± 0.3 ns (500 ± 77 MHz) and completely arbitrary phases. After 18 cycles (36 ns), the ROs reached locking with a steady-state phase skew of 114 ps. Any disturbance on chip, such as supply spikes and charge injection on the ROs phases, directly affects the attained steady state. Although open-loop TDCs cannot recover from such disturbances, the proposed approach is self-regulated by the local feedback from neighboring TDCs, allowing continuous phase/frequency locking. In order to simulate this effect, 32 of the coupled 16×16 array nodes were injected with a disturbance that corresponded to 33% of the overall node charge, after 25 clock cycles, in their most sensitive phase—zero-crossing (see Figure 6a). The process of re-synchronization started immediately after the disturbance, taking about seven clock cycles (14 ns) to reach steady state once again (the same phase skew as before the injection). Figure 6b shows similar simulation, but for a capacitive coupling of $C_{c}$ = 240 fF. After steady state was reached (31 clock cycles), 32 ROs were disturbed with 33% of the total nodal charge. The process of re-synchronization took about 20 clock cycles to return to steady state.

**Figure 7:**
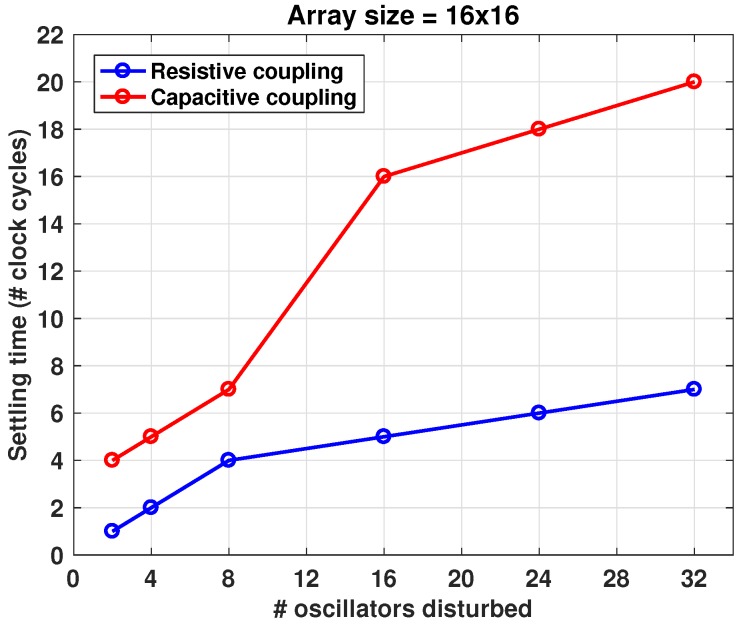
Steady state recovery time (in cycles), after different number of ROs disturbed.

The settling time can vary based on the number of ROs disturbed, the size of the array, and coupling strength. Figure 7 shows this dependency, over a number of disturbed oscillators for the cases of resistive and capacitive coupling.

**Figure 8:**
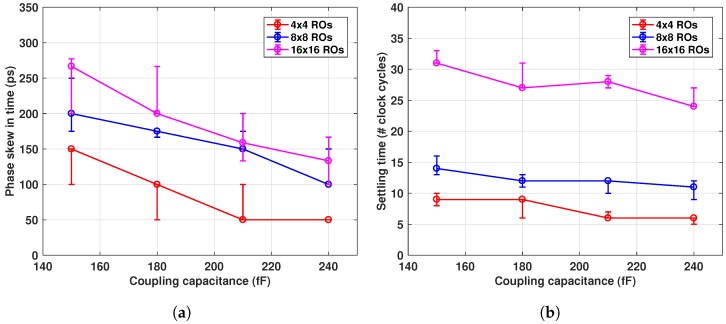
(**a**) Steady state phase skew and (**b**) Settling time for different network sizes and coupling capacitance. Settling time is defined by the phase mismatch below 1/(67%) of value obtained in (**a**); vertical bars indicate variation due to ±10% mismatch in $C_{c}$.

Frequency mismatches and/or PVT variation directly affect the settling time and phase skew. Variations in the coupling impedance also have an impact on the steady state. Thus, apart from ±15% variation on the RO periods, another ±10% on the coupling impedance was included in the simulations. Simulation results for the case of capacitive coupling are shown in Figure 8.

**Figure 9:**
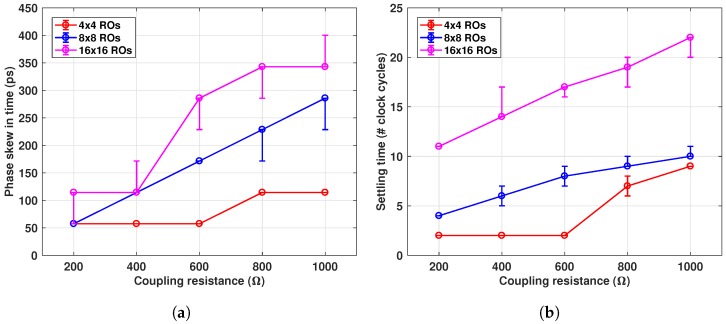
Steady state (**a**) phase skew and (**b**) settling time, for different network sizes and coupling resistance. Settling time is defined by the phase mismatch below 1/(67%) of value obtained in (**a**); vertical bars indicate variation due to ±10% mismatch in $R_{c}$.

The phase skew increased with the number of coupled ROs and for lower coupling impedances. For instance, for the capacitive coupling ($C_{c}$ = 240 fF), it took about six clock cycles for a 4×4 array, to reach steady state, while it took 24 clock cycles for the 16×16 array with the same $C_{c}$, as can be seen in Figure 8b. Similarly, the same steady state parameters were obtained for the case of resistive coupling, as shown in Figure 9. A 600 Ω coupling resistance produced a maximal residual phase skew of 280 ps for the 16×16 array, while for the 4×4, the skew was only 60 ps. Higher coupling resistances also resulted in longer settling time, as shown in Figure 9b.

Charge injection through capacitive coupling only occurs during phase transitions, due to transient voltage variation, which produces longer settling time. Fast coupling is possible by increasing the coupling capacitance. However, due to area constraints and excessive parasitic capacitance, it may limit the overall linearity and operating frequency. Resistive coupling, however, can provide much stronger coupling (lower impedance) at smaller areas, being more suitable for our application.

These results provide a quick insight into the dynamics of mutually coupled ROs, using different types of coupling and different strengths, thus enabling better design choices based on the target application. They also provide a qualitative and quantitative analysis of the synchronization process, allowing better planning for calibration—both foreground and background.

### 3.2. SPICE-Compatible Model

In addition to the macro-model developed in Section 3.1, a SPICE-compatible (based on Verilog-A) model was also used, since electronic circuits are normally designed and simulated in such environments and the interaction with other signals on the readout integrated circuit (ROIC) can be evaluated.

**Figure 10:**
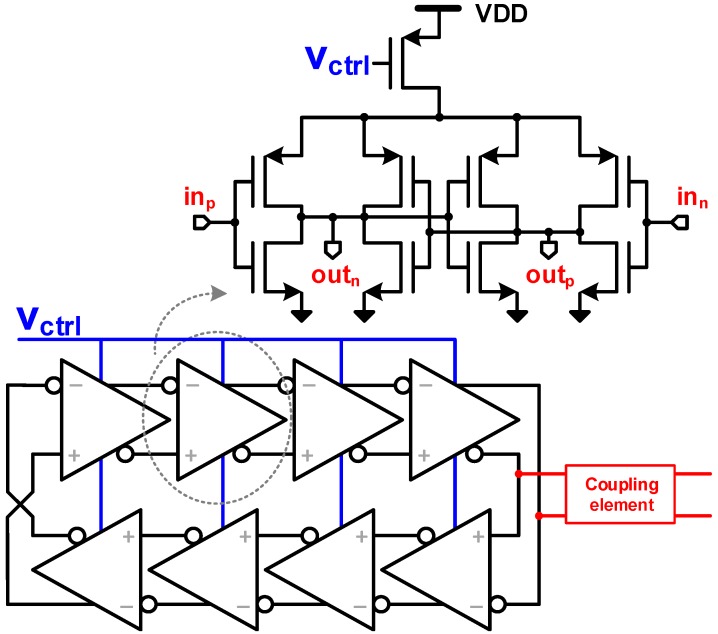
Current-starved 8-stage pseudo-differential RO.

The mode comprises a large-signal differential transconductance, coupled to a capacitive impedance to form each stage of the oscillator [30]. The frequency is controlled by a current source (current-starved RO) and it includes noise effects (thermal and flicker) that are naturally up-converted during oscillation. Although this model can be adapted to different numbers of stages and topology, it was designed to match the RO implemented and measured in Section 4, which is composed of an 8-stage pseudo-differential topology, as shown in Figure 10.

Apart from synchronization, the uncorrelated noise between ROs is filtered out. On average, ROs have low power efficiency—figure of merit (FOM) [31]—on the order of 145–160 dB, which relates their noise (phase noise/jitter) and power consumption. For example, without any elaborate filtering, a 500 MHz RO, consuming 400 μW, and FOM of 150 dB, produces an integrated root mean square (RMS) jitter [32] of about 110 ps (1–100 MHz integration window), which is prohibitively large for millimetric precision measurements, requiring feedback loops for noise filtering at the expense of power, area, and complexity. However, by coupling multiple oscillators, the uncorrelated noise among them is filtered out, providing a reduction in phase noise (and jitter) at the system level by $10\cdot \log_{10}M$ [33], where *M* is the number of coupled oscillators. Although the FOM of the system remains the same (overall power consumption increases and the noise reduces *M* times), at each oscillator, the FOM appears to improve also by $10\cdot \log_{10}M$, with negligible extra power consumption.

**Figure 11:**
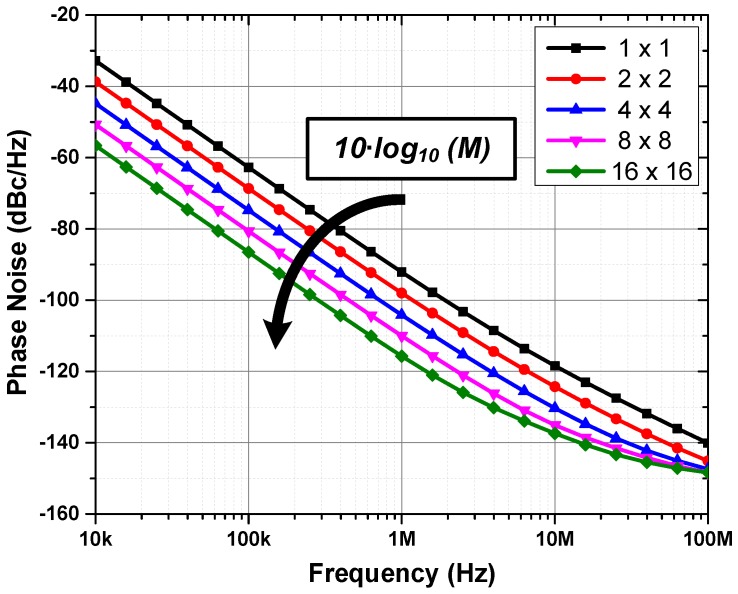
Simulation of phase noise reduction from 1 (1×1) to 256 (16×16) mutually-coupled ROs.

To demonstrate the described effect, multiple oscillator array sizes were coupled, and the simulation result is depicted in Figure 11. The phase noise reduction of the uncorrelated noise (low offset frequencies) behaved as predicted. For the correlated noise (high offset frequencies), such as the thermal noise on the coupling elements, the benefit of the coupling was reduced. A comparison between full SPICE and Verilog-A models was also evaluated. The latter took only 1.5% of the computational power and simulation time of the former, at equivalent precision, providing an essential tool for full chip co-simulation.

**Figure 12:**
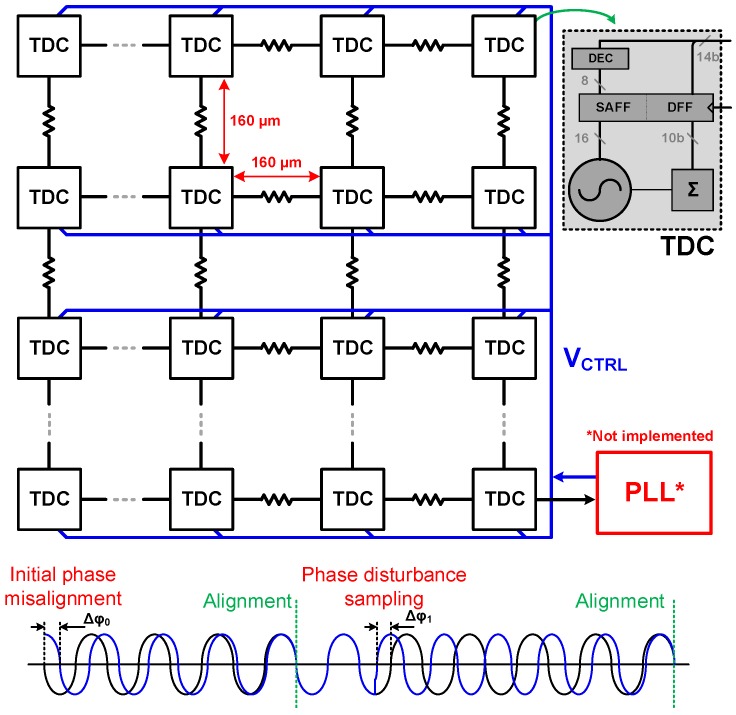
Implemented 8×8 mutually-coupled TDC architecture and RO phase misalignment self-correction. PLL: phase-locked loop.

The implemented block diagram can be seen in Figure 12. Due to resistive coupling, the phase/frequency locking operates on the array at all times, and as a result, both at startup, when the ROs have arbitrary phases (and perhaps different average frequency), or during any disturbance in one or more of the ROs, the array will always be pushed back to a locked state. This is represented by the phase diagram at the bottom of Figure 12. Additionally, due to the nature of the operation and the fact that all ROs are synchronized and share a common control voltage ($V_{CTRL}$), a single PLL can be implemented to define the overall frequency and to track PVT variations, using a single regional phase as reference for the feedback loop.

Thus, starting from the same 150 dB FOM RO at 0.5 GHz (Section 3) and coupling 64 ROs (in an 8×8 structure), the effective FOM was improved by $10\cdot \log_{10}M\approx$ 18 dB, to a moderate 168 dB FOM, which produced an integrated RMS jitter (1–100 MHz) of only 13.75 ps, instead of 110 ps as previously found. For the final topology, an eight-stage, current-starved, pseudo-differential RO was implemented [34].

**Figure 13:**
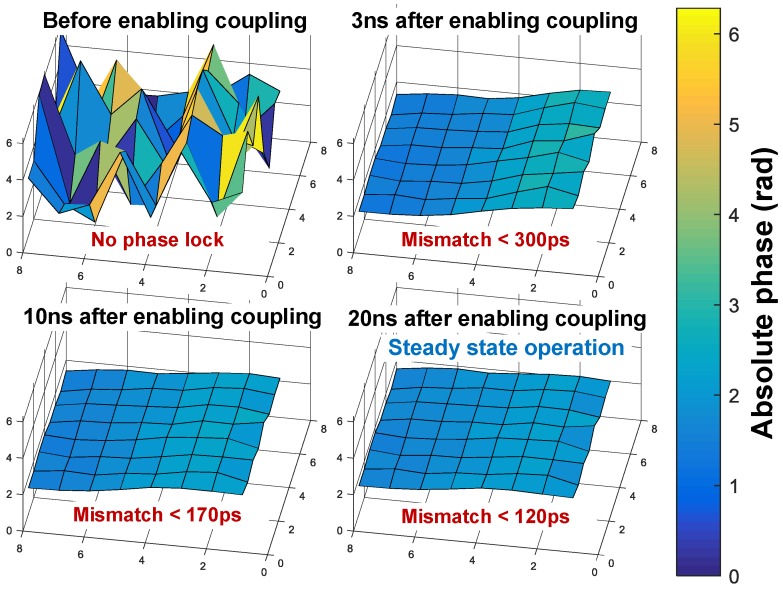
Instantaneous phase mismatch progression, for ±10% RO period variation over the implemented 8×8 TDCs.

The locking process was simulated including ±10% random period variation among the ROs, as in Section 3.1. The variation was performed by introducing a mismatch in the transconductance of each RO. The phase offset in steady state over time is shown in Figure 13, which was less than 1 LSB after 10 oscillation periods for a coupling resistance of 400 Ω.

**Figure 14:**
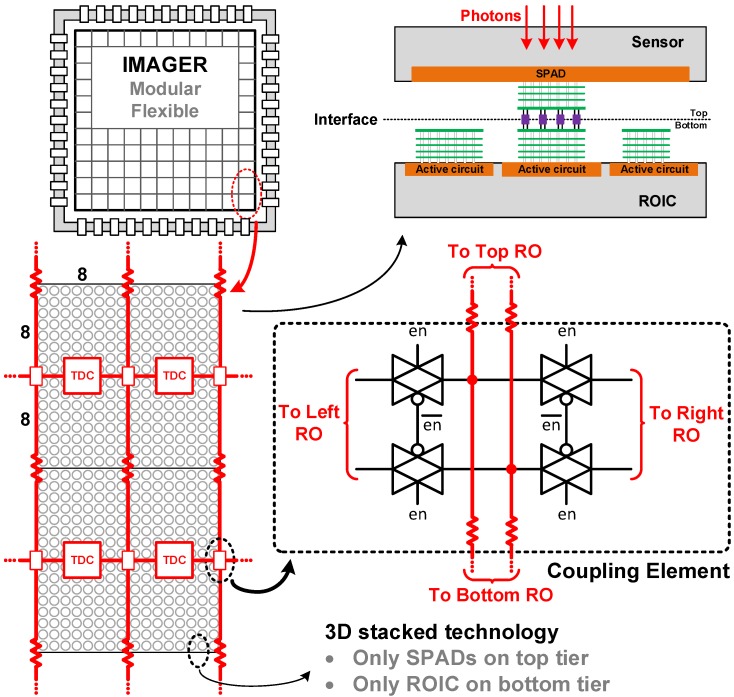
Transmission gate as resistive coupling element. 3D stacked technology implementation.

Along with the RO, a 10-bit ripple counter and D-type and sense-amplifier flip-flops complete the TDC. Based on Section 2, a single TDC was expected to be shared among two independent groups of 8×8 pixels, as sketched in Figure 14. The resistive coupling used was implemented through a transmission gate, shown in Figure 14, so the performance in both modes could be compared. Moreover, it can be used to disable the coupling during initial calibration phase, where all ROs can be adjusted to roughly the same frequency, before coupling, thus improving INL and power efficiency.

## 4. Results

The prototype was fabricated using a 3D-stacked CMOS technology [35], as sketched in Figure 14. The 64 ROs were arranged in an 8×8 matrix, only on the bottom tier, which used low-power, 4 metal (3 thin + 1 thick) 65 nm TSMC technology, with 1.2 V core supply. The proposed technique is independent of the technology and transistor node, also suitable for monolithic implementation, but because the top tier was placed over the TDC array, a chip micrograph could not be obtained.

**Figure 15:**
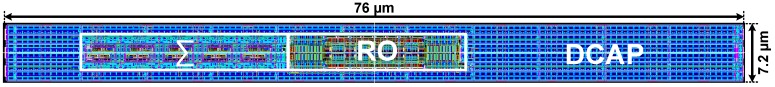
TDC layout.

Coupled and uncoupled conditions were implemented and measured. To mimic the distribution in a real sensor, the TDCs were placed with a pitch of 160 μm, horizontally and vertically, thus achieving a total area of 1.3×1.3 mm${}^{2}$. Each TDC occupied an area of 76×7.2 μm${}^{2}$, including RO, a 10b counter, sampling latches, and decoupling capacitors, which occupied 60% of the TDC array, whose layout is shown in Figure 15.

The effects of the coupling were investigated by measuring the high-frequency clock from the ROs. All 64 ROs were combined through multiplexers and carefully routed to a single high-speed output, connected to a Rohde & Schwarz FSUP-50 signal source analyzer or a Keysight Infiniium DSOS804A real-time oscilloscope for spectrum and phase noise or jitter measurements, respectively.

**Figure 16:**
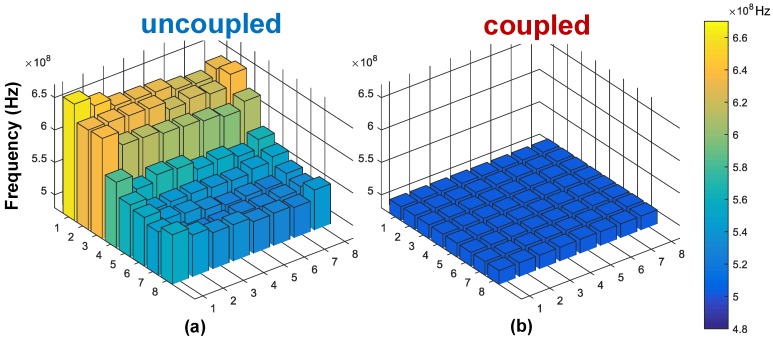
Individual frequencies for different modes: (**a**) uncoupled; (**b**) coupled.

A large IR-drop was present in our fabricated chip because only a few metal layers (3 thin + 1 thick) were available. Its effects on frequency variation can be seen in Figure 16a. Although the intrinsic frequency of each RO varied substantially (about 24%), the mutual coupling was very robust, reaching frequency locking as shown in Figure 16b. Ideally, the ROs should be independently tuned to roughly the same frequency (which can be done by foreground calibration), to ease the process of frequency correction, power consumption reduction (less charge exchange between oscillators), and local INL minimization.

**Figure 17:**
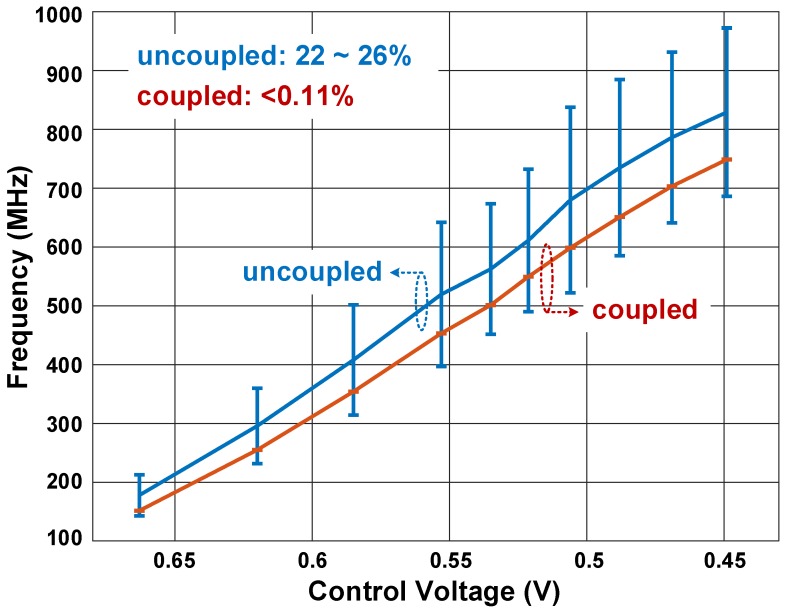
Frequency variation of coupled and uncoupled modes, for different average frequencies.

The array was measured in the whole range of frequencies, from 150 to 800 MHz. The mean values and variation bars, in coupled and uncoupled modes, are plotted in Figure 17. Before coupling, the spread in the instantaneous frequency was 22–26%, whereas under mutual coupling, this spread reduced to less than 0.11%. Moreover, under coupling and, consecutively, locking, all ROs operated in the same average frequency.

It is pertinent to observe that after coupling, the operating frequency was lower than the average of the individual oscillators, both in Figure 16 and Figure 17. The reason is the effect of parasitic capacitance from the coupling element and lines, which was only visible when coupling was enabled. For that reason, the RO was designed with asymmetric stages (stronger for the coupling phase), thus maintaining overall linearity when coupled.

The main goal of this work was to provide an alternative for timing generation and acquisition in large arrays of dTOF sensors. In order to reduce calibration (often difficult to implement in a real application) and resolution uncertainties throughout the sensor, the injection locking technique produced by the mutually coupled oscillators was proposed. However, this technique did not improve the linearity of the individual TDCs, and in fact traded resolution uncertainty for short-range INL.

**Figure 18:**
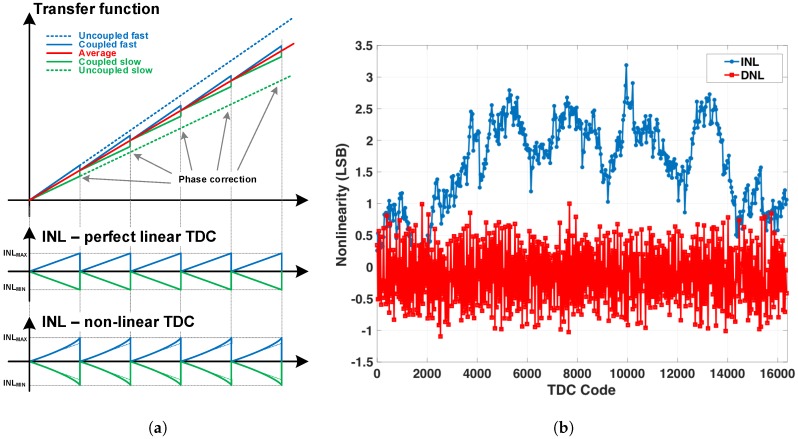
TDC non-linearity effects: (**a**) Local INL due to phase correction, for a perfect linear TDC and a non-linear TDC; (**b**) Uncoupled TDC INL and DNL, without calibration.

For instance, if all TDCs in the array had the same performance (the same RO frequency), by coupling them, they would present the same non-linearity as an uncoupled TDC. However, if variations were present (IR-drop, PVT variations, mismatch, etc.), they would still be locked in frequency and phase, as demonstrated in this paper, but the necessary phase alignment would cause an abrupt non-linearity, increasing the overall INL. An example phase correction is presented in Figure 18a. For an ideal case of perfectly linear TDC, but with different speed, at every RO period the phase needs to be aligned, generating a local INL whose maximum and minimum would depend on the RO period difference to the average period ($|INL_{MAX|MIN}\left|=\right|T_{RO}-T_{AVG}|$). In the presence of intrinsic TDC non-linearity, $|INL_{MAX|MIN}|$ will be a combination of both effects. An illustration of the local INL is shown in the bottom of Figure 18a.

For these reasons, only the uncoupled TDC non-linearity is presented, which was evaluated using a density test method, and the results are plotted in Figure 18b. The maximum INL and DNL were below 3 LSB and 2 LSB, respectively, over the whole 14 bits of dynamic range, without calibration.

**Figure 19:**
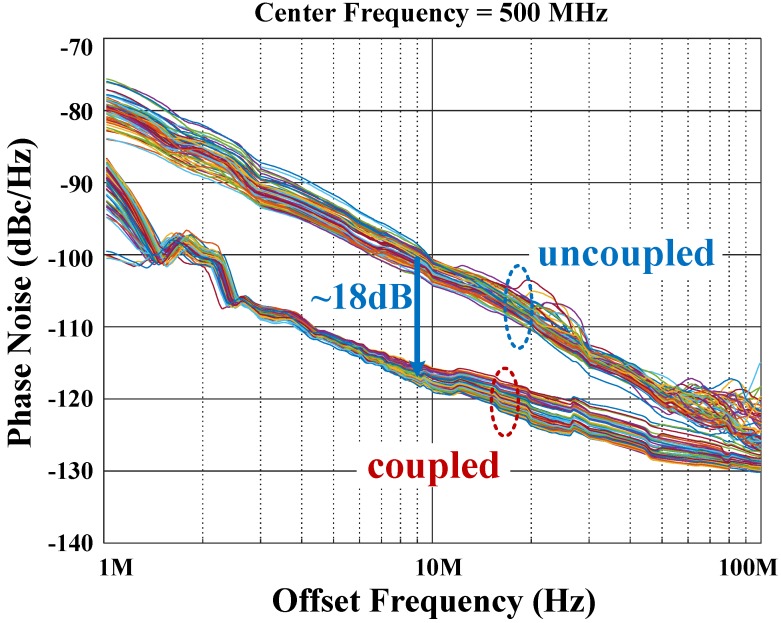
Measured phase noise comparison, for uncoupled and coupled conditions, for all 64 ROs at 500 MHz center frequency.

The phase noise is a key parameter to confirm the effectiveness of mutual coupling on noise filtering and synchronization. Figure 19 shows an 18 dB phase improvement provided by the coupling, for most of the frequency offsets, following the theory. For high-frequency offsets, the coupling elements’ thermal noise dominated the phase noise, and due to its correlation within the array, the coupling was not as effective.

**Figure 20:**
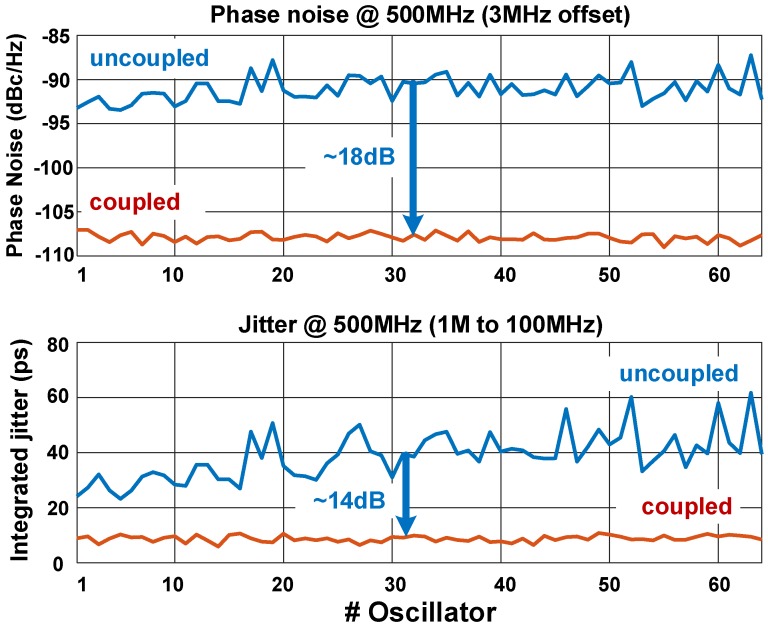
Phase noise and integrated root mean square (RMS) jitter comparison for uncoupled and coupled modes, for all 64 ROs at 500 MHz center frequency.

The phase noise of each RO is plotted along with the integrated RMS jitter in Figure 20. Both measurements were performed with the ROs coupled and uncoupled, at a center frequency of 500 MHz. The phase noise at 3 MHz offset frequency showed the effectiveness of the coupling, reaching an 18 dB improvement on average. The jitter reduction reached 14 dB (instead of 18 dB), due to the presence of correlated noise from the coupling elements.

Figure 16 and Figure 20 show a variation of phase noise and jitter under “uncoupled” mode. The reason being the extreme IR-drop present in the system, where the oscillators close to the edge of the chip (lower indexes, starting from #1) had lower impedance to the supply, and their pMOS current source had higher drain–source voltage, allowing stronger inversion, and thus lower noise factor. Although such conditions existed, it did not affect the synchronization and the noise filtering technique proposed here, which was proved by the phase noise and jitter under “coupled” mode. Nevertheless, the integrated RMS jitter reduction, from about 40 ps to less than 9 ps, was enough for our application, which contained other sources of noise (e.g., SPAD timing jitter [35]) that were much higher.

## 5. Conclusions

Generating a uniform timing reference, used to capture telemetry and depth maps of large arrays of dTOF detectors is very challenging. Constraints on power consumption, area, and technology (e.g., limited number of metal layers for proper power distribution) are some of the key limiting factors. Traditional approaches such as PLL/DLL are not typically applicable (due to area limitation and complexity), whereas column-wise arrangements [7,15] and per-pixel TDCs [4,21,36] are limited to small arrays and photon-starved mode, respectively.

In this paper, we analyzed and compared event-driven to an always-on shared TDC topology, with respect to power consumption and area. From our investigation, supported by a systematic theoretical analysis and by a solid-state implementation, we conclude that for most applications with moderate/high activity, the shared and sampled approach has better power efficiency, with slightly lower saturation of the sensor—especially for short illumination bursts.

Moreover, the always-on TDC array allows uniform and (almost) constant power consumption throughout the sensor, independent of the activity, removing the IR-drop uncertainty typical of event-driven systems. A phase calibration can be performed to compensate residual skew, while PVT tracking is possible through a single PLL, using any phase in the array as reference, since all ROs will be synchronized. The proposed architecture also provides an automatic, fast, and local feedback, where disturbances in the phase of a particular RO are corrected by its neighbors, thus providing a robust, scalable approach to synchronization.

A careful study of the coupling element (resistive/capacitive) was performed and coupling sensitivity was discussed, as was its implication for the settling time and phase error. In general terms, and also intuitively, the stronger the coupling, the more quickly the array reaches steady state, but the more sensitive a TDC is to its neighbors in the case of disturbances.

## 6. Patents

Oscillator arrangement for time-to-digital converter for large array of time-of-flight image sensor devices (Application 15/941,411, 30 March 2018).

## Abbreviations

The following abbreviations are used in this manuscript:

dTOF	Direct time-of-flight
PLL	Phase-locked loop
DLL	Delay-locked loop
TDC	Time-to-digital converter
RO	Ring oscillator
LSB	Least mean square
DNL	differential nonlinearity
INL	integral nonlinearity
SiPM	Silicon photomultiplier
TCSPC	Time-correlated single-photon counting
TTTR	Time-tagged time-resolved
SNR	Signal-to-noise ratio
FOV	Field of view
AR	Augmented reality
VR	Virtual reality
PET	Positron emission tomography
FLIM	Fluorescence lifetime imaging
LiDAR	Light detection and ranging
ADAS	Advanced driver-assistance system
AV	Autonomous vehicles
FIFO	First-in-first-out
RMS	Root mean square
SPAD	Single-photon avalanche diode
PVT	Process-voltage-temperature
ROIC	Readout integrated circuit

## Appendix A

The total power consumption to generate a timing reference, on-chip, can be generically given by a composition of the PLL power consumption ($P_{PLL}$), including all necessary reference buffers, etc., and the dynamic power used on the distribution of multiple PLL phases, thus to be used as fine resolution for interpolative TDC. The number of phases and the frequency will depend on the system architecture. Normally, multiple phases are distributed and used as reference for the local TDCs, in both column-wise [15] and per-pixel TDC approaches [21]. The power consumption associated with the reference is given by:

(A1) $$P_{T}=P_{PLL}+\#p\cdot C_{line}\cdot V^{2}\cdot F.$$

An event-driven TDC starts to operate upon the arrival of a photon, and it is stopped by the end of the time frame. Instead of providing a time-frame value, which can be the inverse of the laser frequency ($F_{laser}$) or shorter, we prefer to define the power consumed by a certain TDC based on its duty cycle. In order to do that, two parameters were created: $\bar{\alpha }$ and $\bar{\beta }$. The former provides an average time a particular TDC stays on, whenever it operated. The latter defines the activity rate, normalized to the laser frequency ($F_{laser}$). For instance, in the absence of noise, $\bar{\alpha }$ will carry a value that positions the target within the time frame, while in a noisy environment, $\bar{\alpha }$ tends to 0.5 (which is the mean value of a uniform variation, such as the uncorrelated noise). On the other hand, $\bar{\beta }$ is defined depending on how many events occurred per laser time frame (which can be larger than 1). If a TDC is shared among *M* pixels, the compounded activity ($\bar{\beta }\cdot M$) should be used, limited to 1 (the TDC can only be activated once per time frame). Thus, the total power consumption over *N* TDCs within the sensor is given by:

(A2) $$P_{TDC}=\bar{\alpha }\cdot P_{TDC}\cdot N\cdot min\left(\bar{\beta }\cdot M,1\right).$$

Finally, in case a single TDC is shared, the power consumption necessary to process such events will depend on the absolute compounded activity of *M* pixels ($\beta \cdot M\cdot F_{laser}$), limited by the dead time of the combination circuit (τ) and the energy consumed for each event propagation, such as:

(A3) $$P_{COMB}=E_{comb}\cdot N\cdot min\left(\bar{\beta }\cdot M\cdot F_{laser},\tau^{-1}\right).$$

The total power consumption is then given by the combination of (A1)–(A3), as:

(A4) $$\begin{array}{cc} P_{T}=P_{PLL}+\#p\cdot C_{line}\cdot V^{2}\cdot F & +\bar{\alpha }\cdot P_{TDC}\cdot N\cdot min\left(\bar{\beta }\cdot M,1\right)\\ & +E_{comb}\cdot N\cdot min\left(\bar{\beta }\cdot M\cdot F_{laser},\tau^{-1}\right). \end{array}$$

Based on the assumptions and conditions described on Section 2.1, and comparing the power consumption of per-pixel and shared, sampled TDC from (2) and (4),

(A5) $$\begin{array}{cc} P_{T,per-pixel} & ⩾P_{T,shared\_sampled},\\ \bar{\alpha }\cdot P_{TDC}\cdot M\cdot min\left(\bar{\beta },1\right) & ⩾P_{TDC}+E_{comb}\cdot min\left(M\cdot F_{laser}\cdot \bar{\beta },\tau^{-1}\right),\\ \bar{\alpha }\cdot P_{TDC}\cdot M\cdot min\left(\bar{\beta },1\right) & ⩾P_{TDC}+M\cdot E_{comb}\cdot min\left(F_{laser}\cdot \bar{\beta },{\left(M\cdot \tau \right)}^{-1}\right),\\ M & ⩾\frac{1}{\bar{\alpha }\cdot min\left(\bar{\beta },1\right)-\left(\frac{E_{comb}\cdot min\left(F_{laser}\cdot \bar{\beta },{\left(M\cdot \tau \right)}^{-1}\right)}{P_{TDC}}\right)}. \end{array}$$
